# Creation of EmpowerMe Website to Promote Self-Efficacy in Survivors of Stroke: Co-Design Study

**DOI:** 10.2196/76756

**Published:** 2026-03-12

**Authors:** Elizabeth A Lynch, Adrian O'Malley, Zoe Adey-Wakeling, Billie Bonevski, Dominique A Cadilhac, Saran Chamberlain, Leonid Churilov, Robyn Clark, Richard Cullen, Erin Godecke, Gillian Harvey, Fiona Jones, Natasha A Lannin, Stacy Larcombe, Jarrad Law, Annette McGrath, Lisa Murphy, Katie Nesbitt, Karly Zacharia, Niranjan Bidargaddi, Coralie English

**Affiliations:** 1 Caring Futures Institute, College of Nursing and Health Sciences, Flinders University College of Nursing and Health Sciences Flinders University Adelaide Australia; 2 Lived Experience of Stroke Sydney Australia; 3 Flinders Medical Centre Adelaide Australia; 4 Flinders Health and Medical Research Institute College of Medicine and Public Health Flinders University Adelaide Australia; 5 Stroke and Ageing Research, Department of Medicine School of Clinical Sciences Monash University Melbourne Australia; 6 Public Health and Health Services Research, Stroke and Critical Care The Florey The University of Melbourne Melbourne Australia; 7 Lived Experience of Stroke Adelaide Australia; 8 Melbourne Medical School The University of Melbourne Melbourne Australia; 9 National Stroke Foundation Melbourne Australia; 10 Stroke Recovery and Rehabilitation Perron Institute for Neurological and Translational Science Perth Australia; 11 Allied Health Research Sir Charles Gairdner Osborne Park Health Care Group Perth Australia; 12 Population Health Research Institute City St George’s University of London London United Kingdom; 13 Department of Neurosciences School of Translational Medicine Monash University Melbourne Australia; 14 Alfred Health Melbourne Australia; 15 Flinders Digital Health Research Centre College of Medicine and Public Health Flinders University Adelaide Australia; 16 CareMappr Adelaide Australia; 17 Lived Experience of Stroke Port Elliot Australia; 18 School of Health Sciences University of Newcastle Australia Newcastle Australia; 19 Heart and Stroke Program Hunter Medical Research Institute Newcastle Australia; 20 Centre for Research Excellence to Accelerate Stroke Trial Innovation and Translation The University of Sydney Sydney Australia

**Keywords:** stroke, website, self-efficacy, co-design, digital health

## Abstract

**Background:**

Digital health innovations are frequently used to support people in managing chronic health conditions. Stroke is common, and people who have survived a stroke and live in the community must learn to manage their health independently. Digital tools can help, but only if designed to match survivors’ specific needs. In response to a need expressed by people living with chronic health conditions, the Australian government created a funding stream to support the development of a digital resource to help individuals gain confidence in managing their health.

**Objective:**

This study aimed to co-design a digital resource to promote self-efficacy to manage life after stroke in community-dwelling survivors of stroke.

**Methods:**

Co-design methodology, which emphasized meaningful engagement with intended end-users throughout the design of the website, was used. The project steering group comprised health professional researchers, digital designers, people with lived experience of stroke (survivors and carers), and representatives from the Stroke Foundation (Australia). A systematic review was conducted to inform the core components of the resource. A lived experience workgroup was convened to advise on features of the digital resource and aspects of its evaluation. Iterative review stages and frequent consultation between the steering group and the lived experience workgroup occurred. Online and in-person usability testing was conducted with survivors and carers.

**Results:**

The lived experience workgroup (workgroup) initially comprised 14 survivors and 1 carer. In total, 11 survivors and 1 carer remained engaged throughout the co-design period. One workgroup member was invited to join the steering group and co-facilitated all co-design workgroup meetings. Defining features of the digital resource were identified by the steering group and the workgroup, including that the resource would be a website that augmented (not reproduced) existing resources, and it needed to be accessible to survivors of stroke with communication changes. Website specifications were determined by the workgroup and included that information needed to be tailored to the individual user, and specific accessibility features were recommended. The workgroup prioritized what content to include on the website and recommended the creation of video stories by Australian survivors and carers. A resource-tailoring tool was created so information could be individualized to the interests of the website user. Overall, 9 web pages containing high-priority content were created, comprising text, video stories, and a downloadable PDF that summarized key information for that page. More than 150 short video stories were created by 26 survivors of stroke and 10 carers for the website. Usability testing indicated that the website was more usable than 83% of all websites.

**Conclusions:**

Authentic co-design with inclusion of people with lived experience of stroke at all stages of development enabled the successful build of a digital resource (website) to improve self-efficacy. An evaluation of the website is underway.

**Trial Registration:**

Australia New Zealand Clinical Trials Registry ACTRN12624001018505; https://anzctr.org.au/Trial/Registration/TrialReview.aspx?id=388255

## Introduction

Chronic health conditions are the leading causes globally of death and disability [1,2]. Effective ways to support people to manage their chronic health conditions have long been recognized as important to maximize health, quality of life, and reduce health care use [3,4]. As populations have become more familiar with using the internet to find health advice [5,6], and advances have been made in available digital health technologies [7,8], there has been a concurrent growth in digital health care delivery, which expanded exponentially with the COVID-19 pandemic [9,10]. Accordingly, digital health innovations are now commonly used to support people to manage their chronic health conditions [11,12].

In 2023, stroke was the second most common cause of death and disability worldwide [1,2]. Despite reductions in the incidence of stroke in high-income countries, the prevalence of people living with stroke is increasing because of aging populations and better survival rates [13]. Affecting more than 100 million people worldwide [14], stroke can affect an individual’s physical ability, energy levels, mood, as well as their ability to communicate, concentrate, and understand [15]. Changes to cognition, communication, and stroke-related fatigue (all affecting at least half of all survivors) [16-18] can present difficulties for survivors in accessing digital health technologies [19,20]. Further, survivors of stroke with communication difficulties and people with cognitive changes are frequently excluded from participating in research [21,22], and little guidance exists in the published literature on how to work with survivors of stroke when designing a digital health innovation.

People living with chronic health conditions were consulted about research priorities to address in Australia and prioritized the development of digital resources to promote health self-efficacy [23]. Self-efficacy is an individual’s confidence in their abilities [24], and when teamed with health, it relates to an individual’s confidence in their ability to manage their health effectively. A research team was convened and sought to develop a digital resource to build health self-efficacy in survivors of stroke.

For any digital innovation, it is recommended that designers work with intended users during the development phase to optimize acceptability and uptake [25]. A co-design approach was chosen so survivors of stroke and other key stakeholders could be meaningfully involved and engaged throughout the entire research process [26], from conceptualizing and building the digital resource, through to its subsequent evaluation. This study describes how the team co-designed an accessible digital resource to promote self-efficacy in survivors of stroke.

## Methods

### Overview

**Figure 1:**
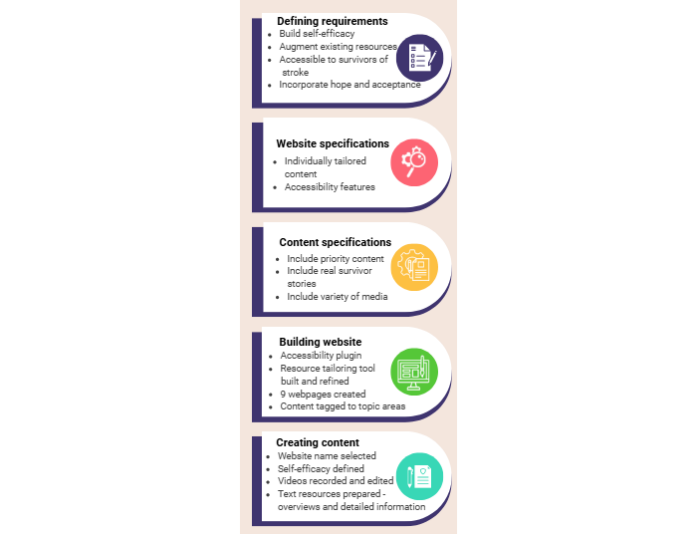
Steps followed to design the EmpowerMe website and content.

The principles of design science in information systems research were used to guide the development of an innovative, purposeful digital resource [27]. The design process was planned to systematically incorporate the needs and preferences of survivors of stroke and carers through each planning and design stage of the digital resource [26]. In total, 4 steps were followed: determining what was required from the resource, determining specifications of the resource, building the resource to the specifications, and testing the resource to determine if it met requirements. These are presented in Figure 1.

### Formation of the Steering Group

**Figure 2:**
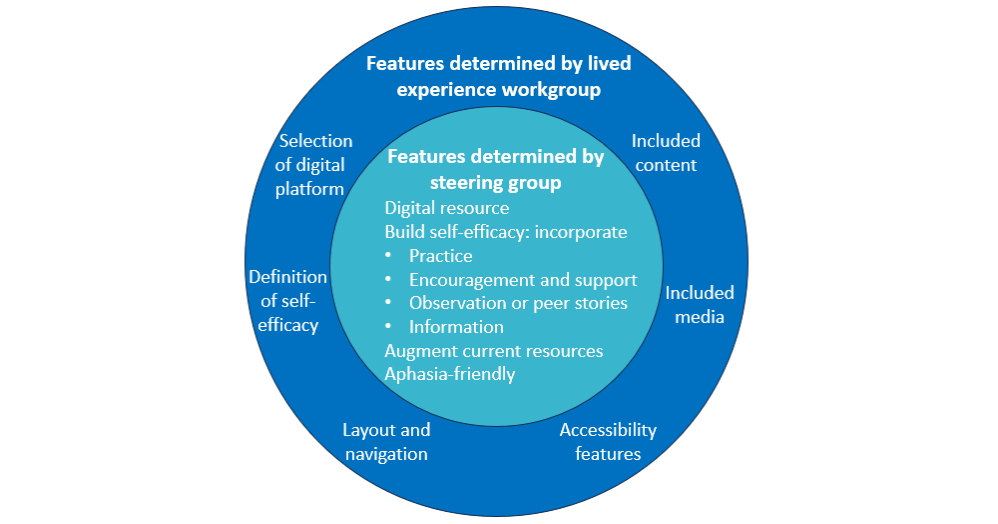
Core and adaptable features of the digital resource.

The team that developed the research proposal and coordinated the study (hereafter referred to as the steering group) comprised 20 members (16 women), invited by Author EAL for their complementary expertise relevant to the project. The steering group included 14 academic researchers from health, bioinformatics, and biostatistics disciplines, 2 survivors of stroke, 1 carer, health professionals, and representatives from Stroke Foundation (Australia), the national stroke advocacy body. The steering group determined the features of the digital resource that were essential, and the adaptable features that should be determined by people with lived experience of stroke (Figure 2). The steering group met online monthly and had oversight of the resource design, timelines, and budget management.

### Formation of Lived Experience Workgroup

A lived experience workgroup (hereafter referred to as the Workgroup) was convened, comprising survivors of stroke and carers, to advise on the adaptable features of the digital resource. Workgroup members were recruited by advertising expressions of interest via social media (Facebook [Meta] and X [formerly Twitter]). Interested individuals contacted a project team member (KZ), who then met online to explain and answer any questions about the project and discuss skills each individual could contribute to the project, in addition to their lived experience. Written information was provided about the project, and all Workgroup members provided informed consent to contribute their ideas and have online meetings video-recorded. Overall, 14 survivors and 1 carer (10 women) were recruited to the workgroup, representing community-dwelling people of different ages (ranging from 43 to 76 years), stroke-related changes, and time since stroke (6 months to 17 years at project commencement). Workgroup members were from 6 Australian states or territories. Some had extensive experience in research and co-design, whereas 3 had no prior research experience. Over the course of the 12-month co-design phase, 3 workgroup members withdrew due to overseas travel, illness, and being too busy.

### Lived Experience Workgroup Meetings

Workgroup meetings were run online via Microsoft Teams and recorded. All Workgroup members were offered support to familiarize themselves with Microsoft Teams at the start of the project. Meetings were co-facilitated by an academic researcher (author EAL) and a survivor of stroke (authors SC or AO’M). Discussion points with or without preliminary reading were sent to attendees before each meeting. Each meeting was attended by a maximum of 5 workgroup members to allow time for contributions from each attendee, so 3 meetings (attended by different workgroup members) were held to discuss each topic.

At the start of each meeting, EAL presented relevant background to the intended discussions and the anticipated outcomes from each meeting. Author SC then facilitated discussions according to the predetermined discussion guide, inviting each person to contribute their ideas. Workgroup members were invited to “think big” and to challenge the status quo when discussing the digital resource design. At the conclusion of each meeting, EAL verbally summarized the key points raised in discussion, and Workgroup members provided agreement or clarification. Discussion points from 1 meeting were presented at subsequent meetings attended by different Workgroup members, to further explore these ideas and determine consensus or conflict. Meeting recordings were reviewed by author EAL, and key points of discussion, consensus, or conflict were documented and distributed to all attendees within a week of the final meeting. Attendee names were not included in meeting summaries. Attendees were invited to provide further details if they considered that information was missing. Facilitators SC and EAL shared key points from workgroup meetings at the bi-monthly steering group meetings, where final decisions about the digital resource design were made (in all instances, Workgroup recommendations were approved). Workgroup members were remunerated for their time at rates recommended by the New South Wales Consumer Alliance.

Overall, 6 rounds of workgroup co-design meetings were conducted between June and December 2023, interspersed with 3 online surveys between meetings, which followed up on workgroup discussions regarding content to include and how to measure the efficacy of the developed digital resource.

### Online Surveys

Survey 1 comprised 19 questions, and Workgroup members were asked to rate the importance of including content about 18 different health-related needs (identified from the Australian Stroke Needs Assessment Survey [28]) using a 5-point Likert Scale (definitely include; probably include; consider including; probably DO NOT include; definitely DO NOT include). An additional question allowed free-text responses seeking any other information to include. In Survey 2, workgroup members were asked to rate the importance (using a slider from 0 not at all important to 100 extremely important) of measuring 28 items related to self-efficacy that were identified in Workgroup discussions and from the Australian Stroke Needs Assessment Survey [28]. Respondents were also asked to nominate their top 3 items from the included list, and a final question allowed free text for any additional comments to consider about measuring the efficacy of the digital resource. In Survey 3, 2 shortlists of 10 items from Surveys 1 and 2 were presented. Workgroup members were asked to rank items in order of importance regarding the priority content to include and the priority aspects of self-efficacy to measure.

### Survey Data Analysis

Data from Surveys 1 and 2 were analyzed descriptively. The frequency of “definitely include” responses for items in Survey 1 was calculated, and the means and frequency in the “top 3” were calculated for each item in Survey 2. Data from ranking exercises in Survey 3 were analyzed using a graph theory-based voting system [29], wherein group-based ordinal rankings were generated from the individual ordinal rankings.

### Determining Technological Features

**Figure 3:**
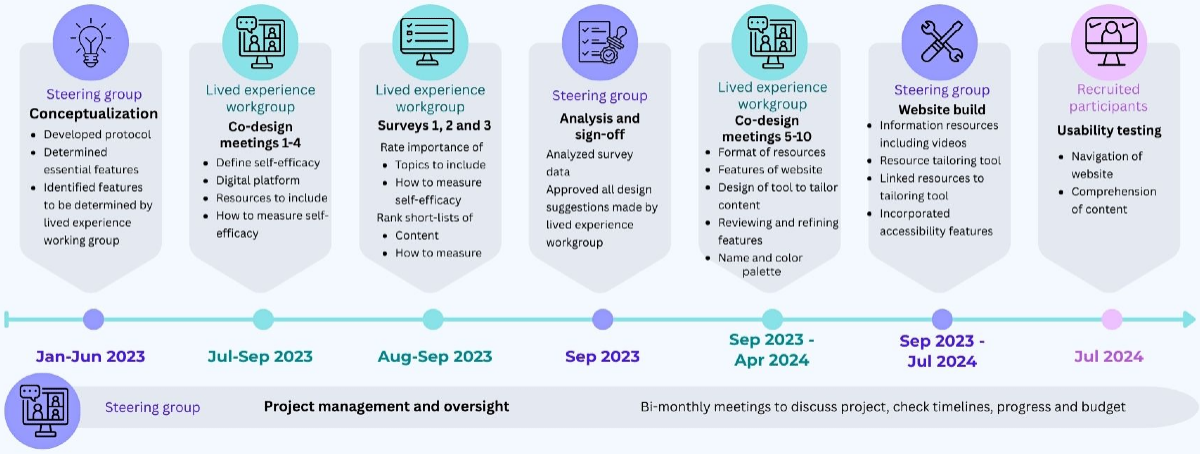
Timeline of website co-design and usability testing.

A further 3 rounds of co-design meetings with the Workgroup were co-facilitated by a digital designer and a survivor of stroke (author SC) to determine the technological features of the digital resource. The workgroup was reconvened between March and April 2024 to review the digital prototype. The topics discussed at the meetings are presented in Table 1. The project timeline is illustrated in Figure 3.

**Table 1:** Focus of workgroup meetings.

Meeting number	Main agenda items
1	Introduction to the project Discuss ways of working Definition of self-efficacy Personal experience of building self-efficacy after a stroke
2	Terms of reference Definition of self-efficacy Do different groups of people have different self-efficacy needs? Resources to support self-efficacy Selection of a digital platform
3	Measuring self-efficacy Content to include on website
4	Content to include on website Existing resources that should be included Preferred format Previous (good and bad) learning experiences using digital technology
5	Recap of progress to date Format of resources Features to include on website
6	Experiences using websites for information about health or well-being Features of websites that make them harder or easier to use, more or less enjoyable Opinions on Interactive features on websites Sharing personal information on websites Receiving “push” emails or text messages Any changes to the way information is processed since the stroke
7	Identifying an individual’s information-seeking behaviors and digital preferences
8	Reviewing the prototype of the quiz to tailor content
9	Reviewing and refining the prototype of the quiz
10	Reviewing website prototype Feedback on layout and color palette Name of website

### Usability Testing

The tailoring of usability testing procedures and methods to meet the needs of people with language and cognitive changes has been presented elsewhere (Manuscript submitted to *Journal of Medical Internet Research* concurrently). A total of 3 groups of participants were recruited for usability testing: survivors of stroke with no self-reported language changes (n=4), survivors with moderately severe aphasia (a language disorder that affects communication; n=4), and carers of survivors of stroke (n=3). None had been involved in the design or development stage. Participants were offered an Aus $50 (US $35.45) gift voucher on completion of the testing session to thank them for participating.

### Usability Testing Procedures

Testing was conducted in a one-to-one moderated format by author KN, with support from author EG for participants with aphasia. In-person testing was organized for all participants with aphasia, and in-person or online participation was offered to other participants. Usability scripts were prepared to guide participants to test navigation around the website and evaluate comprehension of the content (Multimedia Appendix 1).

### Data Collection

Demographic data, time taken to complete tasks (for each task and total cumulative time), percentage of tasks completed, knowledge acquisition (questions about definition and importance of self-efficacy), System Usability Scale (SUS), and user experience (survey developed by the research team incorporating specifics of the EmpowerMe website, based on the Website Evaluation Questionnaire).

### Usability Data Analysis

Participant demographic data and usability task-time data were analyzed descriptively using IBM SPSS Statistics 28.0.0.0. Knowledge acquisition was analyzed using the chunk and check technique [30,31] and reported narratively. Mean (SD) for overall, individual, and group SUS scores were analyzed using the SUS calculator package [32], and using this package, the raw mean score was normalized and converted to a percentile rank. The percentile rank indicated the relative usability of the website compared to other digital products. Group mean scores for survivors of stroke with and without aphasia were compared using a between-subject 2-sample *t* test (2-tailed), with statistical significance set at *P*≤.05. Data from the user experience survey were analyzed descriptively and tabulated against task-time and task-completion data to examine usability from different perspectives, providing an opportunity to make targeted website changes when issues were identified.

### Ethical Considerations

The project had ethical approval (5886) from Flinders University’s Human Research Ethics Committee. People in the lived experience workgroup provided written informed consent for their contributions to be used to shape the digital resource design and for meetings to be video-recorded. Due to the group nature of workgroup meetings, the ideas generated were not confidential and were shared with other workgroup members. Anonymized summaries were shared with the project steering group. Workgroup members were offered Aus $100 (US $70) per meeting, inclusive of preparation time, paid via invoice or electronic gift cards.

Usability testing participants provided written informed consent to participate. The research speech pathologist (EG) prepared an aphasia-friendly participant information sheet and explained the information to each participant with aphasia, using aphasia-friendly language and allowing time for processing of information. She checked the participant’s understanding of the information as part of the consent process. Data from usability testing participants were collected anonymously to protect participants’ confidentiality, and only the research team had access to the data. Each participant was offered an Aus $50 (US $35) gift card as remuneration for completing the usability testing.

## Results

### Defining Requirements of the Digital Resource

The lived experience workgroup recommended that a website would be the most acceptable digital resource to support survivors of stroke develop their self-efficacy. Essential requirements determined by the Steering Group before project commencement were that the website would be free to access and needed to augment, not reproduce, information and other supports already available (such as available through Stroke Foundation and its website EnableMe [33]). The website needed to be accessible to non-experts in stroke, with low or medium levels of health literacy, and accessible to people with stroke-related changes, including changes to cognition, communication, or reading ability. The Workgroup highlighted the importance of including representation of people of different ages, genders, and cultural backgrounds.

Another essential requirement of the website was that it needed to support self-efficacy in Australian survivors of stroke and people in their support network (family members, friends, and carers), so it should incorporate features such as learning from or observing other survivors and carers, and provide information. Elements that were identified by workgroup members as being important to building self-efficacy and a sense of well-being after stroke were having a purpose in life, having self-compassion, having goals to work toward, and being realistic while also having hope. Acceptance that things have changed and having resilience to bounce back if experiencing a setback were also recognized as intertwined with a person’s confidence in their abilities. These elements guided the development of resources hosted on the website.

### Setting Specifications

#### Specifications of Website

Workgroup members expressed that the website needed to incorporate a feature so that information could be tailored to each user, who could then “choose their own adventure” to find information or other resources relevant to them. Interactivity in the form of a chatbot was universally rejected by workgroup members. However, a feature where users were asked a series of questions about areas of interest or information needs that would then guide the user to a curated list of relevant resources was deemed to be of benefit. The Workgroup also recommended that the tailoring feature be optional, so users who preferred not to provide information about themselves, or people who prefer to “browse,” could look through all the available resources.

In terms of accessibility features for survivors of stroke, the use of consistent layout and spacing was suggested to improve the accessibility of information for people with visual field loss. Use of sans serif font, having aphasia-friendly text options, and availability of captions for videos and adjustable playing speed were recommended to meet the needs of people with communication impairments. The Workgroup recommended having a text-to-voice option so users could choose to listen to rather than read the written information. Use of large boxes for clickable links (rather than underlined text) was recommended to improve functionality for people with motor control difficulties. Avoiding any spontaneous animations, background music, and moderating the tone of voice and volume on videos was important for people who experience sensory overload.

Other recommended features were not specific to the needs of survivors of stroke, including having options to adjust the website colors and having clear navigation pathways.

#### Specifications of Content to Include

Priority content to include on the website was determined by the workgroup following discussions and surveys. The list of priority content topics is presented in Table 2. The most important topics to address were “What is self-efficacy and why is it important?” and “How to be involved in health care decisions.” Due to substantial overlap in content, 2 topics (“Managing concentration problems” and “Managing cognitive changes”) were grouped, leaving 9 priority topics to address.

**Table 2:** Priority content to include and the number of videos created.

Topic number	Topics, prioritized by the lived experience workgroup	Number of videos	
		Survivors	Carers
1	Defining self-efficacy and its importance	50	9
2	Being involved in decisions	7	4
3	Seeking the information you need	10	6
4	Advocating for yourself	12	2
5	Managing fatigue	11	2
6	Emotional problems	10	3
7	Managing cognitive changes^a^	6	3
8	Managing mobility changes	11	2
9	Managing speaking difficulties	10	9
10	Managing concentration problems^a^	—^b^	—^b^

^a^Due to substantial overlap in content, “managing cognitive changes” and “managing concentration problems” were combined into one topic.

^b^Not available.

Results from a systematic review conducted by the research team about self-management interventions to improve self-efficacy in survivors of stroke [34] did not highlight any further components that needed to be incorporated into the website.

#### Specifications of Content Format

To meet different users’ learning styles, the website needed to host content in a variety of media, including video, text (including downloadable information sheets), and audio resources. For written information, having both “bite-size” information chunks and “deep-dive” detailed information for topics was preferred, so a user could browse headlines of topics and then go into detail on topics of interest to them.

To enhance self-efficacy via vicarious learning from peers, the steering group and workgroup planned to include content delivered via testimonials from survivors of stroke and carers. There was a strong preference from workgroup members for these to be real, unscripted video-stories of Australian survivors and carers who shared their struggles and how these were managed, as well as their experiences of recovery. Specifically, Workgroup members recommended using home recordings (eg, via smartphone camera or Zoom videoconferencing) rather than professional videographers to prepare the video stories, as they felt this improved the relatability of the person sharing their story and made the stories and messages more credible. The preferred length of videos was less than 3 minutes.

For the delivery of information prepared by health professionals, Workgroup members preferred to have real people in any videos rather than using graphics or animations, and the use of Australian-specific information when available.

### Building the Website and Developing Content

#### Incorporating Accessibility Features

An accessibility plugin (UserWay) was used to allow users to customize the site to their needs. This included options like increasing contrast, enlarging the text, increasing the text spacing, highlighting links so clickable elements are more prominent, and other options.

#### Building the Resource Tailoring Tool

The Workgroup members met with a digital designer and shared their poststroke information experiences, device usage patterns, content preferences, and strategies for validating health information. Following this session, the designer developed distinct user personas (Table 3) and used this information to develop a quiz-based tailoring prototype to accommodate all identified personas. The subsequent 2 sessions were used to refine the prototype through iterative feedback, emphasizing content relevance and quiz structure. Workgroup members provided critical input on optimal questionnaire length, preferred response formats, and content delivery mechanisms.

Following development and feedback, the resource tailoring tool was trialed and finalized. It comprised 2-3 (depending on responses) multiple-choice questions that subsequently became more precise to identify topics of interest to each user. Additional questions were added to the start of the tool to collect demographic data of users for research purposes (Textbox 1).

**Table 3:** Personas of survivors of stroke accessing digital information emerging from the design sessions.

Characteristics	Tech savvy (n=4)	Traditional learner (n=3)	Supported learner (n=3)
Digital skills	High confidence with multiple devices	Prefers structured content	Confident but needs accessibility features
Learning style	Self-directed research and critical evaluation	Relies on medical professionals	Institution-guided learning
Information sources	Multiple sources, fact-checking, and research background	Printed materials from traditional health channels	Verified institutional sources
Preferred content format	Short videos, blogs, and interactive content	Academic content and structured text	Short videos (<15 min) and articles
Device usage	Multiple devices (phone, tablet, and laptop)	Preference for larger screens	Phone and laptop primarily
Support needs	Independent learners	Guided learning approach	Accessibility adaptations

**Questions to guide content curation. Topic was a high priority and had a web page developed as part of this project.**

**What topics would you like to explore today?**

**How to build my confidence after a stroke**

- Finding the information I need
- Learning from survivors or carers
- Practice
- Support from others
- Skill development

**How to speak up for what I want or need**

- Self-advocacy
- Involvement in decisions about my care

**How to manage the effects of the stroke**

- Managing physical changes
  Using my arm
  Bladder and bowel problems
  Falls prevention
  Mobility changes
  Swallowing
- Managing changes that are hard to see
  Vision
  Cognitive (thinking and memory) changes
  Speaking difficulties
  Pain and sensation changes
  Emotional changes
  Fatigue

The resource tailoring tool was a plugin to the website built using Flutter Web, which sat on top of the website to add the quiz-based tailoring information. The structure of the Tailoring Tool and the content provided were stored in a Webiny Content Management System. Upon using the Tailoring Tool, relevant resources were pushed to website users – see Organizing Resources within the Website section.

#### Preferred Language and Definitions

Terminology was considered and workshopped in both workgroup and steering group meetings. Most workgroup members (except for individuals with occupational therapy or education backgrounds) were unfamiliar with the word “self-efficacy” at the start of the project, and some found it hard to pronounce. Many steering group members and workgroup members felt that the word “self-efficacy” was not part of general day-to-day language and were concerned that use of it might be off-putting. However, some workgroup members felt that it was important to educate survivors and carers about self-efficacy, and familiarization with the word “self-efficacy” was an important educational component that could empower survivors and carers. The Steering Group then decided to use lay language (avoiding the word “self-efficacy”) in headings and titles and include the word “self-efficacy” in second-tier text designed to inform and educate users.

After several iterations, the preferred definition of self-efficacy, determined by the Workgroup, was “Belief in my abilities to achieve the things that matter to me.”

#### Naming the Website

Initially, the Steering group anticipated naming the website ASSET-Stroke according to the project name (derived from co-designing a Digital Solution for Self-Efficacy After Stroke). However, both the steering group and workgroup agreed that this name did not encapsulate the philosophy and purpose of the website and was unlikely to attract the attention of survivors of stroke unfamiliar with the project. Accordingly, discussions were facilitated, and voting was conducted with the workgroup, and the domain name EmpowerMe was selected. This name aligns with others curated by Stroke Foundation, such as EnableMe [33] for survivors of stroke and carers and InformMe [35] for health professionals working with survivors of stroke.

#### Creating Website Content

Project team members created content and accompanying downloadable PDFs to address each priority topic area. All text was edited until it was deemed to be understandable by people who had completed grade 7 at school by Microsoft Copilot. Text resources for “Managing speaking difficulties” were edited by a speech pathologist with expertise in aphasia (author EG) and then checked and further edited with a survivor of stroke with moderate aphasia.

Expressions of interest were advertised for survivors of stroke and carers to create video stories to be hosted on the website about building self-efficacy after stroke, with a focus on the prioritized topic areas. Twenty-six survivors of stroke and 10 carers recorded video stories, usually over Zoom with a project team member, lasting up to 30 minutes. The project team edited the recordings into numerous short (30-s to 3-min) videos, creating more than 150 videos (Table 2). Each edited video was sent to the individual for approval or further editing before uploading to the Stroke Foundation YouTube channel, and being allocated to a topic area (one of the 9 priority topics when appropriate). A thumbnail with a brief title was provided for each video. Video contributors were reimbursed for their time.

#### Creating Web Pages

The main website was developed on WordPress, which allowed content to be easily updated by nontechnical team members, if needed, and was compatible with Stroke Foundation’s systems to allow Stroke Foundation to manage the website on project completion. The 9 priority topics were organized into 9 web pages. The website home page presented information from the highest priority topic, “Defining self-efficacy and its importance.” The resource tailoring tool was presented at the top of the page, presented as a prominent click box to “Find resources relevant to you.”

The subsequent 8 topic web pages were created using a standard template, the use of icons, and formatting to ensure consistent layout. Each page had a lay description of the topic, a video about the topic, text about how to build self-efficacy in the topic, written content for carers about the importance of self-efficacy for carers or the carer’s role in supporting self-efficacy of survivors, and thumbnails and titles of relevant videos created by survivors and carers relevant to the topic.

#### Organizing Resources Within the Website

Resources created as part of the project, and existing resources relevant to the priority content areas, were tagged by project team members regarding topic, format, and weighting in the Webiny Content Management System. Resources developed as part of the project were weighted higher than preexisting resources, and Australian resources were weighted higher than internationally produced resources. High-priority topics (identified during the co-design phase) had whole web pages and embedded resources developed, and the web page received top-weighting for the topic.

On using the resource tailoring tool, a curated list of relevant resources was created for each individual based on their responses regarding their areas of interest and associated resources’ tags and weightings.

### Usability Testing of the Website

#### Navigation of Website: Task Completion (Time Taken and Percentage Completed)

Survivors of stroke without aphasia (communication difficulties) and carers completed their tasks more quickly and completely than survivors with aphasia. Activities where survivors of stroke with aphasia struggled were reviewed by the team, and adaptations were made to enhance accessibility for this group of survivors.

#### Comprehension of Content

Overall, 6 participants (4 survivors with aphasia and 2 survivors without aphasia) participated in the evaluation of comprehensibility of website content. In total, 4 participants (2 [50%] survivors with aphasia and 2 [100%] survivors without aphasia) were able to understand and explain 100% of the content included in the knowledge assessment. One survivor with aphasia completed the “chunk and check” for 1 task only (20% of content included in knowledge assessment), and the remaining survivor with aphasia was unable to complete any knowledge tasks. These 2 survivors had more severe aphasia, so it was deemed that the website content was suitable for survivors with mild-moderate aphasia, and more work was required to meet the needs of survivors with severe post-stroke communication difficulties.

#### SUS

The SUS data were normally distributed, so overall mean scores, group mean scores, and between-subject comparison were calculated (comparing survivors with aphasia to survivors without aphasia, comparing survivors without aphasia to carers). SUS scores of survivors of stroke with aphasia were significantly lower than scores of survivors without aphasia (mean 61.3, SD 6.6 vs mean 86.8, SD 2.4; 95% CI –36.8 to –14.4). There was no statistically significant difference in SUS scores between carers and survivors without aphasia (mean 88.3, SD 11.5 vs mean 86.9, SD 2.4; 95% CI –23.3 to 26.3).

The normalized raw mean SUS score for the website was 78, and following the calculation of the percentile ranking, the usability of the EmpowerMe website was deemed better than 83% of all websites.

#### User Experience

Suggestions to improve the website from participants, which were also noted by the usability session moderator, included improving the visibility of resources after use of the resource tailoring tool; addressing layouts that were difficult for people with poststroke visual inattention; and improving problematic layouts (text heavy) for people with aphasia. These were subsequently addressed by the design team.

### Website Efficacy

The potential of the digital resource “EmpowerMe” to influence self-efficacy, health-related quality of life, participation, and anxiety and depression in different cohorts of survivors (stratified by age and time since stroke) is currently being evaluated as part of a phase II trial.

## Discussion

### Principal Results

We have presented how a digital resource in the form of a website can be built specifically to meet the needs of a client group with heterogeneous challenges, including cognitive, language, and motor changes. Rather than retrofit an existing website to the needs of people with poststroke visual, motor, cognitive, or sensory changes, we designed the website proactively to meet the diverse needs of the intended user group. People with lived experience of stroke were included at all stages, from preparation and submission of the grant, being members of the steering group, to facilitating and contributing in lived experience workgroup meetings and contributing to this article.

The Steering Group actively sought to balance the power dynamic between the academic researchers and people with lived experience. Following project commencement, the team recognized the need for more lived experience leadership to facilitate the lived experience workgroup meetings. One workgroup member (SC, survivor of stroke) with experience in both research and in facilitating online peer groups accepted an invitation to join the steering group, cofacilitate workgroup meetings, and was employed as a research assistant for the life of the project to assist in data analysis.

Within a research framework, we were successfully able to embed systems to support people with a lived experience of stroke to contribute meaningfully alongside a team of multidisciplinary experts to co-design a digital resource to address self-efficacy after stroke. Recognizing the different expertise of each party allowed the team to determine who decided each feature of the digital resource, to simultaneously address the needs and preferences of survivors of stroke and carers, and maintain the rigor and aims of the research process.

Survivors of stroke and carers, who comprised the lived experience workgroup, determined the digital platform (website with a feature to direct the user to individually relevant information), the priority content to include, and the priority format of resources (home-made video stories) to be developed by the project team. In contrast, the project Steering Group (which included 2 survivors of stroke and 1 carer) made fewer decisions but worked consistently to incorporate feedback and suggestions from the lived experience workgroup, positioning these within best-practice frameworks of adult learning, self-efficacy, self-management, and digital design.

### Comparison With Prior Work

There is a growing body of literature documenting how to facilitate co-design and interactive workshops online, particularly since the COVID-19 pandemic [36-39]. Now that social distancing measures have lifted, online methods of co-designing have remained popular, with obvious benefits of reducing travel time, costs, and increasing accessibility for people who live in regional and remote areas [39,40]. While some researchers in previous work have described using interactive features of online platforms to enhance activity within online co-design meetings [37,41], interactions with the lived experience working group in the current project were mostly dialogue-based, although they were augmented with online surveys between meetings, similar to what others have also described [41].

The project reported in this article is far from the first website to have involved intended users in the design. Supporters of collaborative design methods argue that involving target users when designing a digital resource will improve the resource's impact through better user engagement and satisfaction [40,42-44]. However, the level of involvement of the intended users can vary greatly between studies that report using co-design methods. While co-design has been described as “meaningful end-user engagement in research design and includes instances of engagement that occur across all stages of the research process” [26], it is also acknowledged that the engagement activities “range in intensity from relatively passive to highly active and involved [26]. In a systematic review which tabled the most common co-design activities in studies reporting co-design of digital health solutions, 2 of the most common activities reported were interviews and surveys [45]. Implicit in these activities are the research team setting the agenda, seeking a one-way transference of “data” from participants, and controlling how the data are analyzed, which can be disempowering (rather than engaging) for participants [46]. Other commonly reported co-design activities that may promote more engagement were workshops [45], which tend to be more collaborative and allow for a two-way exchange of information, having been described as occasions in which a group of people can acquire new knowledge or perform creative problem-solving [47].

Intended users have been variably involved in determining whether a website would be of benefit, ranging from not being involved in the decision, (for instance when the rationale of developing a website is based on disease incidence and rates of technology use [43]), to contributing data that is used by researchers to support the project idea (eg, rationale is underpinned by interviews of participants living with the condition under investigation [48]) to sharing responsibility for the decision to develop the website (eg, when people with lived experience are members of the research team and help shape the research proposal [49,50]).

Similarly, the level of input and decision-making responsibility of representatives of the intended user group about features of the co-designed websites varies. Intended users could contribute data to be used by the design team to make decisions (such as people being recruited as participants in interviews, focus groups or co-design sessions) [42,48], they could advise on features (eg, via lived experience advisory groups) [42,48,50] or be decision-making partners (eg, via being members of the research or design team) [49,50]. Just as in our project, several studies reported involving people from the intended user group in different roles within the project [42,48,50], for instance, with some people recruited as participants, others consulting in an advisory capacity, and others partnering on the research team. We have advanced the field beyond existing data tools by explicitly working with the intended end users to embed features to tailor content and accessibility to survivors of stroke. Unique to this project was the need to develop a website that was accessible to people with language, visual, motor, cognitive, or sensory changes from their stroke. By proactively incorporating accessibility features into the design (rather than retrofitting these to a standard website design), we anticipate the website will address the variety of challenges faced by survivors of stroke. Further, by co-creating content, the voices and preferences of survivors of stroke have been prioritized to support peer learning and peer support.

Compared to prior work, the current project used more active and involved methods of working with people with lived experience of stroke throughout the design of the website than has been reported in most other publications reporting on co-design of websites to support health or well-being. We identified 1 published work presenting the co-design of a website to support physical activity and diet to reduce secondary stroke [50]. This was also conducted in Australia, with 4 academic researchers and 1 team member from the Stroke Foundation also involved in the current project. These 2 Australian projects involved contributions from people with lived experience of stroke and included survivors of stroke in the authorship team; the current project convened a Lived Experience workgroup, and Pogrebnoy et al [50] consulted people with lived experience via a Consumer Advisory Group (1 survivor of stroke advised on both projects). Differences include that the website developed by Pogrebnoy was an adaptation of a newly created telehealth service, whereas in this study, the program and content were co-designed and integrated as part of the website build. Together, the projects demonstrate that collaborative ways of working are feasible, even when working with people who experience communication, cognitive, or physical challenges after their stroke. These ways of working are transferable to other clinical fields, and the methods reported in this study could be used by other research and design teams.

### Limitations

By nature of engagement, wherein meetings were conducted online via Microsoft Teams, the lived experience workgroup members who contributed over the duration of the project tended to have high levels of digital literacy. This was a proof-of-concept project, and there was no specific strategy to recruit people from linguistically or culturally diverse backgrounds. Several team members (on the Steering group and Lived Experience Workgroup) were born in countries other than Australia, but most team members spoke English as a first language. Should the website indicate promise at improving outcomes for survivors of stroke, future work will be undertaken to engage with culturally and linguistically diverse groups of survivors and carers, and with people with lower digital literacy to determine their interest in accessing and using a website to promote self-efficacy. Partnership with the community leaders would be sought so that similar methods as have been described in this study could be used to understand and address the needs and preferences of groups of people that have not yet been represented on the EmpowerMe website.

### Conclusions

We have demonstrated how a multidisciplinary team of academic researchers, people with lived experience of stroke, and Stroke Foundation representatives can work collaboratively from the inception of a project to co-design a digital resource known as the “EmpowerMe” website, which hosts content to meet the needs of survivors with stroke to support self-efficacy. Our team was convened to answer a question raised by people with lived experience of chronic health conditions, the team comprised people with varied skills and experiences (including people with lived experience of stroke and Stroke Foundation representatives), we convened a lived experience advisory group which we consulted over the course of the project to ensure the resource met the needs of the intended user group. Finally, we had clear roles and responsibilities for the steering group, the lived experience workgroup, and the usability testing participants. In this way, this project can serve as a reference for other co-designed digital health resources and offer practical lessons for similar initiatives.

## Appendix

Multimedia Appendix 1 Usability testing script and list of customizable features.

## Abbreviations

SUS: System Usability Scale

## Data Availability

The datasets generated or analyzed during this study are available from the corresponding author on reasonable request.
