# Supplementary material for: Creation of EmpowerMe Website to Promote Self-Efficacy in Survivors of Stroke: Co-Design Study
Source: J Med Internet Res. 2026 Mar 12;28:e76756. doi: 10.2196/76756 (PMC13022539; doi:10.2196/76756)
Supplement: Multimedia Appendix 1 [file jmir_v28i1e76756_app1.docx]

**Supplementary File 1. Usability testing script**

**Usability session script (task)**

| **Hypothesis** | **Carer task** |
| --- | --- |
| Find self-efficacy video on landing page | *Go to information on self-efficacy* |
| Locate and watch video on self-efficacy | *Open, enlarge, watch, reduce video on self-efficacy* |
| Complete resources tailoring tool | *Find resources that would be helpful to you* |
| Open a specific resource | *Read one of the resources* |
| Navigate back to the website | *Read another resource* |
| MOVE TO SCENARIO BASED TASKS | |

**Usability session script (knowledge)**

| **Hypothesis** | **Survivor/carer task** | **Outcome *(facilitators teach back questions)*** |
| --- | --- | --- |
| Find self-efficacy video on landing page | *Go to information on self-efficacy* |  |
| Locate and watch video on self-efficacy | *Open, enlarge, watch, reduce video on self-efficacy* |  |
| CHUNK AND CHECK | | "I want to make sure the website explains things clearly and in a way that is interesting. If you will tell me what is self-efficacy?  Why is it important to build this after having a stroke? |
| Complete resources tailoring tool | *Find resources that would be helpful to you* |  |
| Open a specific resource | *Read one of the resources* |  |
| CHUNK AND CHECK | | “You have gone over a lot of information, and I want to make sure it is clear to you. So, tell me, what do you think are the three most important things to know about what you have just read? (this will depend on what resource they read) |
| Navigate back to the website | *Watch the video on practice* |  |
| CHUNK AND CHECK | | It important to us that this information is clear for people using the website. Can you describe how practicing a task builds self-efficacy? |
| Navigate to the topics, choose one, read/watch/listen  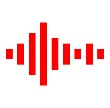 TEST READ ALOUD FUNCTION HERE | *Choose a topic that is interesting or relevant to you*  Imagine you have a sight problem…now listen to the page you are on |  |
| CHUNK AND CHECK | | Has this information built your self-efficacy, and if so, can you tell me how? |

**Supplementary File 2. List of Customizable features**

| Contrast | Increases contrast between foreground and background colours – helpful for people with low vision or colour blindness |
| --- | --- |
| Screen Reader | Reads text on screen aloud |
| Smart Contrast | Automatically adjust contrast based on content and background to ensure optimal readability |
| Highlight Links | Website links highlighted to increase visibility |
| Bigger Text | Allows for larger font |
| Text Spacing | Allows user to adjust line height and spacing between words and letters |
| Pause Animations | Allows animations to be paused – helpful for people with vestibular changes, visual inattention, sensory overload |
| Hide Images | Allows user to hide images – useful if using a screen reader |
| Dyslexia Friendly | Design and selection of font that reduces clutter, reduces cognitive load of people with dyslexia (and others) |
| Cursor | Customisations that assist users with visual and motor impairments |
| Tooltips | Message or tip when user hovers over an interactive website element |
| Page Structure | Consistent page alignment to support users with aphasia or visual disturbances to read |
| Line Height | Sufficient line spacing to improve readability |
| Text Align | Aligned to left for easier reading |
| Saturation | Allows saturation adjustment - higher for people with visual impairment or reduced saturation for people with photosensitivity |
| Dictionary | To support comprehension of included text |
